# Neurotherapeutics for ADHD: Do they work?

**DOI:** 10.1002/pchj.544

**Published:** 2022-03-31

**Authors:** Katya Rubia

**Affiliations:** ^1^ Department of Child & Adolescent Psychiatry/PO46, Institute of Psychiatry, Psychology & Neurosciences King's College London London UK

**Keywords:** attention‐deficit/hyperactivity disorder (ADHD), brain stimulation, fMRI‐neurofeedback, functional magnetic resonance imaging (fMRI), near‐infrared spectroscopy (NIRS)‐neurofeedback, transcranial direct current stimulation (tDCS), transcranial magnetic stimulation (TMS), trigeminal nerve stimulation (TNS)

## Abstract

This paper reflects on the use of neurotherapeutics for attention‐deficit/hyperactivity disorder (ADHD). ADHD is the most imaged child psychiatric disorder, with over 3 decades of magnetic resonance imaging (MRI) research. Findings are relatively homogeneous compared to other psychiatric conditions with consistent evidence for differences, albeit small, relative to healthy controls in the structure and function of several frontal, parietotemporal, and striatal brain regions as well as their inter‐regional structural and functional connections. The functional deficits have been targeted with modern neurotherapeutics, including neurofeedback (using most commonly electroencephalography and more recently functional near‐infrared spectroscopy and functional MRI) and non‐invasive brain stimulation (such as repetitive transcranial magnetic stimulation, transcranial direct current stimulation, or external trigeminal nerve stimulation). Except for electroencephalography‐neurofeedback, the majority of neurotherapeutic studies have been relatively small, with very heterogenous research protocols and outcome measures and—likely as a consequence—inconsistent findings. Furthermore, most brain stimulation studies have tested effects on cognitive functions rather than clinical symptoms. So far, findings have not been very promising. Future studies require systematic testing of optimal protocols in large samples or homogenous subgroups to understand response prediction that could lead to individualized treatment.

## INTRODUCTION

Attention‐deficit/hyperactivity disorder (ADHD) is defined in the *Diagnostic and Statistical Manual of Mental Disorders* (5th ed.; American Psychiatric Association, 2013) as a disorder of persisting and impairing symptoms of age‐inappropriate inattention, and/or hyperactivity/impulsivity. It is one of the most common childhood disorders with a worldwide prevalence of around 7% (Thomas et al., 2015) and persists into adulthood in most cases where it is associated with comorbidities and poor social and academic outcomes (Thomas et al., 2015).

People with ADHD commonly have problems with so‐called “executive functions,” which are higher‐level cognitive functions necessary for mature adult goal‐directed behaviors, such as motor response inhibition, working memory, switching, sustained attention, and intraindividual response variability (Pievsky & McGrath, 2018), as well as with timing functions (Noreika et al., 2013; Rubia et al., 2009). These executive functions are mediated by late developing fronto‐striato‐parietal and fronto‐cerebellar networks (Rubia, 2013), which are typically underfunctioning in children and adults with ADHD compared to healthy controls during the resting state and when performing tasks that target these functions (for review see Rubia, 2018). For example, several functional magnetic resonance imaging (fMRI) meta‐analyses have shown cognitive domain‐dissociated underactivations in several inferior and dorsolateral prefrontal, striatal, parietal, and cerebellar brain regions in ADHD (Cortese et al., 2012; Hart et al., 2012, Hart et al., 2013; Lukito et al., 2020; McCarthy et al., 2014; Norman et al., 2016). ADHD patients have also been shown to have abnormally increased activation in areas of the default mode network (Hart et al., 2012, 2013), which consists of intercorrelated activation of the ventromedial frontal cortex, posterior cingulate, precuneus, inferior parietal and temporal regions and is thought to reflect task‐irrelevant thoughts (i.e., mind wandering; (Raichle, 2015). The combination of decreased activation of task‐relevant regions and decreased deactivation of the default mode network reflecting more mind‐wandering has been suggested to be responsible for the poor performance in ADHD on attention‐demanding higher‐level cognitive tasks (Rubia, 2018).

Clinical treatment is most successful with psychostimulant medications, which enhance catecholamines in the brain, with an effect size of ~0.8 for parent‐ratings of symptoms and about 70% of patients with ADHD responding to it (Cortese et al., 2018). Second‐line treatment is with noradrenaline transporter/receptor blockers atomoxetine and guanfacine, which also enhance brain catecholamines with effect sizes of 0.56 and 0.67, respectively (Cortese et al., 2018). ADHD medications, however, commonly have side‐effects and while very effective in short‐term randomized controlled trials, longer‐term efficacy over several months or years has not been demonstrated in meta‐analyses, or observational or epidemiological studies (Cortese et al., 2018; Swanson, 2019). A meta‐analysis of positron emission tomography (PET) and single photon spectroscopy (SPECT) studies as well as a prospective PET study showed that long‐term medication is associated with an upregulation of dopamine transporters which could potentially suggest brain adaptation (Fusar‐Poli et al., 2012; Wang et al., 2013). Furthermore, parents and children prefer non‐pharmacological treatments (Ferrin et al., 2012; Waschbusch et al., 2011) and in particular have high hopes for transcranial direct current stimulation (tDCS) as a short‐term treatment given the low side‐effects they have experienced (Buchanan et al., 2022).

## NEUROTHERAPEUTICS IN ADHD

One of the most revolutionary neuroscience findings over the past two decades has been the discovery that the brain is extremely plastic, not only in the developing period, but also in adulthood (Draganski et al., 2004). There is indeed a bidirectional relationship between brain and behavior where not only brain injuries can cause behavioral changes, but experience can shape the brain. For example, several weeks or months of training of a particular skill in adults, such as juggling (Draganski et al., 2004), learning for an exam (Draganski et al., 2006), or meditation (Dodich et al., 2019), can change the structure of underlying brain regions. These insights into the neuroplastic potential of the brain have led to an exponential increase in the testing of novel neuromodulation treatments—such as non‐invasive brain stimulation or neurofeedback—as clinical interventions. The pediatric population is even more susceptible to neuroplastic changes induced by neuromodulation due to the developmental neuroplasticity.

Functional MRI studies of ADHD over the past three decades have provided meaningful targets for neurotherapeutics. It seems plausible that neurotherapies that target these key neurofunctional abnormalities could improve the disorder. Electroencephalography (EEG)‐neurofeedback has been tested for over 46 years, with the latest meta‐analyses showing small and some non‐significant findings (Riesco‐Matias et al., 2021). FMRI‐Neurofeedback has been conducted so far with too few studies and very small‐numbered samples in ADHD to provide meaningful evidence. Non‐invasive brain stimulation studies have been conducted in relatively small numbers with highly heterogenous study designs and consequently inconsistent findings with respect to improving cognition and little evidence, so far, on improving clinical ADHD symptoms.

### Neurofeedback

Neurofeedback is based on operant conditioning that teaches participants to volitionally self‐regulate specific regions or networks using trial and error through real‐time auditive or visual feedback of their brain activation. The feedback is typically represented on a PC in the form of a thermometer or with a videogame for children to make it more attractive. EEG‐neurofeedback has been examined in ADHD for over 45 years. There are 10 meta‐analyses reviewing the evidence, with the latest meta‐analysis showing small to medium effect size of superiority of EEG‐neurofeedback compared to non‐active control groups for improving parent‐rated ADHD symptoms and for improving the inattention subdomain for teacher ratings; however, effects are inferior to pharmacotherapy (Riesco‐Matías et al., 2021).

Real‐time fMRI neurofeedback enables participants to self‐regulate the blood‐oxygen level‐dependent response of a targeted brain region or network through real‐time feedback of their brain activity. Functional MRI‐neurofeedback has the advantage of superior spatial resolution compared to EEG‐neurofeedback and it can target the key cortical and subcortical brain function deficits that have been established in ADHD over the past 26 years of fMRI research (Rubia, 2018). Functional MRI‐neurofeedback has shown some promise in improving clinical symptoms and cognition in other psychiatric disorders (Thibault et al., 2018). To date, however, there are only two published fMRI‐neurofeedback studies in ADHD. A small randomized controlled trial in 13 adults with ADHD asked patients to do a mental calculation task with (*n* = 7) and without (*n* = 6) fMRI‐neurofeedback of the dorsal anterior cingulate in four weekly scans of 60 min (Zilverstand et al., 2017). Both groups significantly increased anterior cingulate activation but did not differ in improvements in ADHD symptoms observed in the two groups at trend level. However, only the neurofeedback group showed significant improvement in sustained attention and working memory tasks, suggesting some positive effects of fMRI‐neurofeedback of the dorsal anterior cingulate on cognition (Zilverstand et al., 2017). A randomized controlled trial from our lab tested fMRI‐neurofeedback of the right inferior frontal cortex (rIFC) compared to fMRI‐neurofeedback of the left parahippocampal gyrus in adolescents with ADHD (Alegria et al., 2014). Thirty‐one boys with a clinical ADHD diagnosis had 4 hour‐long scans over 2 weeks, in which they did 11 runs of 8.5 min of fMRI‐neurofeedback with a rocket movie as feedback. Eighteen participants learned to self‐upregulate the rIFC, while 13 participants self‐upregulated a control region, the left parahippocampal gyrus. In both groups, activation of their respective target regions increased progressively across the 11 fMRI‐neurofeedback runs. However, only the rIFC‐neurofeedback group showed a transfer effect (self‐regulation without feedback, as a proxy of transfer to real life) that correlated with reduced ADHD symptoms. There were no group differences in ADHD symptom improvements after the treatment, but both groups improved. However, only the rIFC‐neurofeedback group showed a large ADHD symptom reduction at the 11‐month follow‐up, with an effect size of almost 1, compared to an only trend‐level reduction in the left parahippocampal gyrus‐neurofeedback group. Only the rIFC‐neurofeedback group also showed trend‐level improvement in a sustained attention task. The rIFC‐neurofeedback group also showed increased functional connectivity between the rIFC and the anterior cingulate cortex and caudate, and a decrease in functional connectivity between the rIFC and regions of the posterior default mode network. These connectivity findings suggest that not only the targeted region improved in activation but so did entire networks that are connected to this region (rIFC; Rubia et al., 2019). To assess the effects of fMRI‐neurofeedback on brain function in ADHD, the participants also performed a motor response inhibition fMRI task before and after treatment. The rIFC‐neurofeedback relative to the left parahippocampal gyrus‐neurofeedback group showed increased activation after compared to before neurofeedback in the rIFC and parietal regions during inhibition (Alegria et al., 2014) and increased activation in left‐hemispheric IFC/insula and striatal regions during performance monitoring, which correlated with ADHD symptom improvements and better performance (Criaud et al., 2020). The increases of activation in the IFC and striatal regions were similar to those we observed previously with stimulant medication (Rubia et al., 2014), suggesting that fMRI‐neurofeedback of the rIFC has similar brain upregulation effects. Last, there were no group differences in side‐effects or adverse events. However, when we tested neurofeedback learning capacity, we found that only 48% of patients learned successfully to upregulate their target region with fMRI‐neurofeedback, which is similar to the EEG‐neurofeedback literature (Lam et al., 2020). The best predictors of fMRI‐neurofeedback learning were not clinical or cognitive data but enhanced fronto‐striatal activation in the fMRI Stop task at baseline (Lam et al., 2020).

The only pilot study that tested near‐infrared spectroscopy (NIRS)‐neurofeedback trained upregulation of the left dorsolateral prefrontal cortex (DLPFC) in 11 hour‐long sessions over 4 weeks in nine ADHD children and compared it with EEG‐neurofeedback (*n* = 9) and electromyography‐neurofeedback (*n* = 9). Only NIRS‐neurofeedback showed significant improvements in clinical ADHD symptoms and in performance in inhibition and attention functions, which was, however, not superior to EEG‐ or electromyography‐neurofeedback (Marx et al., 2015).

In conclusion, fMRI‐neurofeedback and NIRS‐neurofeedback research is still very new with only two small studies in children. Some of the within‐group improvement findings of these small proof‐of‐concept studies are promising. However, larger, double‐blind, placebo‐controlled randomized controlled trials are needed to more thoroughly assess the potential efficacy of these neurotherapies in ADHD.

### Brain stimulation

Non‐invasive brain stimulation therapies, specifically repetitive transcranial magnetic stimulation (rTMS), transcranial direct current stimulation (tDCS), and trigeminal nerve stimulation (TNS), have been applied to ADHD only very recently, over the past decade. These stimulation techniques are thought to influence cellular and molecular mechanisms involved in use‐dependent local and distant synaptic plasticity, that is, GABA and glutamate‐mediated long‐term potentiation, which may lead to longer‐term brain plasticity (Demirtas‐Tatlidede et al., 2013). Studies in healthy adults and different patient populations have shown up to 1 year longer‐term cognitive effects after stimulation with rTMS or tDCS (Rubia et al., 2021; Westwood, Radua, & Rubia, 2021a).

Furthermore, there is evidence that both techniques can lead to increased levels of catecholamines (Rubia et al., 2021; Westwood, Radua, & Rubia, 2021a), which are known to be abnormal in ADHD (Cortese et al., 2018). The “electroceutical theory” of neurostimulation suggests that nascent biochemicals (such as dopamine and noradrenaline) are enhanced by the electrical stimulation, which can alter the activity of communication between specific nerve fibers to achieve therapeutic effects, while the “augmentation theory” of neurostimulation suggests that therapeutic benefits arise from physicochemical means, such as changes to the transmembrane potentials, membrane permeability, or electroactivity of receptors or receptands, under the influence of the applied electric field (Camp et al., 2021). For both rTMS and tDCS it seems that the combination with cognitive training, which primes the areas to be stimulated with a cognitive task, is more effective than stimulation alone, due to the synergistic effects of functional targeting (Westwood, Criaud, et al., 2021b; Westwood, Radua, & Rubia, 2021a). Trigeminal nerve stimulation has been applied in ADHD only relatively recently, and indirectly can activate fronto‐striato‐thalamic systems via stimulation of the brain stem (Rubia et al., 2021).

### Repetitive transcranial magnetic stimulation

Repetitive TMS is a relatively safe non‐invasive brain‐stimulation technique that uses brief, intense pulses of electric currents delivered to a coil placed on the subject's head in order to generate an electric field in the brain via electromagnetic induction. Typically, high‐frequency rTMS promotes cortical excitability, while low‐frequency rTMS inhibits cortical excitability. Repetitive TMS has greater specificity in targeting neural regions than tDCS, but is more expensive and more painful, which makes it less suited for children. Most common side‐effects are relatively minor and are transient, such as temporary scalp discomfort underneath the coil due to stimulation of the pericranial muscles and peripheral nerves (Westwood, Radua, & Rubia, 2021a).

The majority of rTMS studies with two exceptions were conducted in adults with ADHD. Six studies applied between one and 25 rTMS sessions of 20–30 min duration. Two double‐blind, sham‐controlled crossover studies stimulated the right DLPFC. One session of 20 Hz‐rTMS relative to sham significantly improved overall self‐rated ADHD symptoms and inattention in 13 ADHD adults but had no effect on hyperactivity (Bloch, 2012). Ten daily sessions of 10 Hz‐rTMS relative to sham in nine ADHD adults had no effect on self‐rated clinical symptoms adults, EEG measures, or cognitive performance (Weaver et al., 2012). A single‐blind sham‐controlled randomized study showed no effect on self‐rated clinical or cognitive measures of sustained attention in 22 ADHD adolescents after 20 daily sessions over 4 weeks of 18 Hz deep rTMS over bilateral DLPFC (*n* = 13) compared to sham (*n* = 9; Paz et al., 2018). A parallel, semi‐blind randomized, active and sham‐controlled study of 15 sessions over 3 weeks of 18 Hz‐rTMS of both DLPFC and IFC—combined with a short cognitive training session before and after stimulation—and a 1‐month follow‐up maintenance session in 43 ADHD adults found significant improvements in ADHD symptoms (Alyagon et al., 2020). No significant effects were observed on other clinical, cognitive, and EEG measures, but EEG measures under the stimulation area correlated with clinical symptom improvements. In children with ADHD, the first, open label tolerability and safety trial (*N* = 10) of five daily sessions of 1 Hz‐rTMS over the left DLPFC showed fewer teacher‐rated inattention and parent‐rated hyperactivity/impulsivity symptoms 1 week after treatment compared to baseline (Gómez et al., 2014). The second pediatric study in 60 children with ADHD found that 30 daily sessions of 25 min of 10 Hz rTMS over the right DLPFC over 6 weeks combined with atomoxetine, compared to atomoxetine (1.2 mg/kg) alone or rTMS alone, significantly improved ADHD symptoms but no other clinical or cognitive measures (Cao et al., 2018). Both pediatric studies did not include a sham condition, however, and hence placebo effects cannot be excluded for the improvements within groups. With respect to safety, the majority of studies reported no side‐effects or serious adverse events other than most commonly transient itching or headache under the stimulation site.

In conclusion, with the exception of one larger randomized control trial (RCT) that stimulated both DLPFC and IFC combined with cognitive training, but which needs replication, there is relatively little evidence that several sessions of rTMS over a frontal site improve ADHD symptoms or cognition. However, studies were relatively underpowered and conducted relatively few session numbers of rTMS with only two studies in children without a placebo condition.

### Transcranial direct current stimulation

In tDCS, scalp electrodes apply a weak, relatively painless and persistent direct electric current to underlying brain regions with the current passing between a positively charged anode and a negatively charged cathode. Anodal stimulation leads typically (but not always) to an increase, and cathodal stimulation to a decrease (cathodal stimulation) of the excitability of underlying neurons via the generation of subthreshold alterations of neuron membrane potentials that modify spontaneous discharge rates; this can increase or decrease cortical function and synaptic strength. Compared to TMS, tDCS is much easier to apply, cheaper, and less painful and hence more suitable for children. Side‐effects are minimal and typically transient, such as itching and reddening of the scalp site of stimulation in some people (Westwood, Radua, & Rubia, 2021a). Currents are typically applied for 20 min in one session, which can be combined with a cognitive paradigm, which can boost the effect (Westwood, Radua, & Rubia, 2021a).

The majority of tDCS studies (13 out of 18), unlike the rTMS studies, were conducted in children rather than adults with ADHD, presumably due to the high tolerability and low side‐effect profile. Most studies applied one to five sessions of about 20 min of tDCS in children or adults with ADHD, with the exception of our study, which applied 15 sessions. Only four studies tested for clinical symptoms, three studies after five sessions of tDCS of DLPFC and one study after 15 sessions of tDCS of right IFC; two studies in nine and 15 ADHD patients, respectively, found an improvement with real compared to sham tDCS on clinical inattention symptoms, which persisted 1 or 2 weeks later (Cachoeira et al., 2017; Sotnikova et al., 2017). One study found an improvement with transcranial random noise stimulation (tRNS) of left DPFC and right IFC compared to tDCS of left DLPFC combined with cognitive training on ADHD symptoms in 19 patients (Berger et al., 2021). However, the largest study, which tested 15 sessions of tDCS of right IFC in 50 ADHD patients, found no improvement compared to sham in clinical symptoms and even an improvement with sham relative to tDCS (Westwood, Criaud, et al., 2021b). All other studies tested the effects of tDCS on a range of executive cognitive functions and found an improvement on some but not other functions (Westwood, Radua, & Rubia, 2021a) with little consistency in findings between studies, and few of them correcting for multiple testing. Two meta‐analyses tested the effects of tDCS on cognitive performance in ADHD. The first meta‐analysis included 10 studies, including a total of 201 children/adults with ADHD and found that one to five sessions of anodal tDCS over mainly left DLPFC significantly improved cognitive performance in inhibition measures (Hedges' *g* = 0.12) and in n‐back reaction times (*g* = 0.66; Salehinejad et al., 2019). However, effect sizes were small and the meta‐analysis did not control for interdependency between measures likely overestimating statistical significance, and included attention measures within the inhibitory measures (Westwood, Radua, & Rubia, 2021a). Our larger meta‐analysis of 12 tDCS studies, including a total of 232 children and adults with ADHD, found that one to five sessions of anodal tDCS over mainly left DLPFC led to small and only trend‐level significant improvements in cognitive measures of inhibition (*g* = 0.21) and of processing speed (*g* = 0.14), but not of attention (*g* = 0.18; Westwood, Radua, & Rubia, 2021a). In conclusion, the findings of the use of tDCS to improve ADHD symptoms and cognition are mixed, with only four studies testing for clinical effects and meta‐analyses showing some positive results on improving cognition, with, however, very small effects sizes.

Far fewer studies stimulated the right IFC. Most studies tested one session and found no significant cognitive improvements (Westwood, Radua, & Rubia, 2021a). We conducted the largest double‐blind, sham‐controlled RCT in 50 children with ADHD, where we found that 15 sessions of 20 min of right IFC stimulation combined with cognitive training in executive function tasks showed no superior effect relative to sham on clinical symptoms or cognitive functions (Westwood, Criaud, et al., 2021b) nor on EEG measures (Westwood, Bozhilova, et al., 2021c). A double‐blind cross‐over study found that five sessions of transcranial random noise stimulation (tRNS) over left DLPFC and right IFC compared to tDCS of left DLPFC combined with executive function training in 19 children with ADHD improved clinical symptoms after treatment and 1 week later as well as working memory and processing speed during sustained attention (Berger et al., 2021). The only study that stimulated the right inferior parietal lobe in 17 ADHD children in a single‐blind crossover study found improved performance in bottom‐up orienting attention but deteriorated selective attention and had no effect on alerting or top‐down executive attention (Salehinejad et al., 2020).

To conclude, there is large heterogeneity in tDCS studies with respect to study designs, stimulation protocols, and outcome measures, which makes it difficult to make consistent conclusions. While relatively safe, the larger studies found no clinical effects with multi‐session tDCS and meta‐analyses show small effects of improving cognition.

### Trigeminal nerve stimulation

External trigeminal nerve stimulation (TNS) is another non‐invasive intervention with minimal side‐effects. TNS transmits small electrical currents transcutaneously via a self‐adhesive, supraorbital electrode to excite (trigger action potentials) the supratrochlear and supraorbital branches of the ophthalmic nerve (V1) located under the skin of the forehead. The supraorbital nerve has widespread connections to the brain, in particular the reticular activation system, locus coeruleus, brain stem, thalamic, frontal and other cortical areas (Shiozawa et al., 2014). It also has effects on catecholamines, which have effects on arousal and attention and have been implicated in ADHD (Cortese et al., 2018; Rubia, 2018). Two studies tested the efficacy of TNS in ADHD, which is typically applied every night for several weeks. An 8‐week, open trial, pilot feasibility study showed significant reduction in ADHD symptoms in 21 children with ADHD, in depression symptoms and in behavioral executive functions in daily life with some positive effects on selective attention and inhibitory control. A subsequent blinded, sham‐controlled proof of concept study of the same authors of 4 weeks of TNS in 62 children with ADHD showed a significant improvement in the active relative to the sham TNS group in ADHD symptoms and trend‐level differential improvement for anxiety but not for depression (McGough et al., 2019). There was furthermore increased EEG activity in the active relative to the sham group in right frontal midline and inferior frontal regions after treatment compared to before, which correlated with the clinical improvements suggesting mediation of the clinical effects (McGough et al., 2019). Both trials showed that TNS was well tolerated with no serious adverse events and relatively minor and transient side‐effects, such as headache or fatigue. Based on evidence from this small, underpowered proof‐of‐concept study, TNS is now the only brain stimulation technique that is approved for ADHD.

## CONCLUSIONS: DO NEUROTHERAPEUTICS WORK IN ADHD?

Modern neurotherapeutics in ADHD is still very much in its infancy. Neurofeedback studies using higher spatially resolved neuroimaging techniques, such as NIRS and fMRI, have only recently been piloted in ADHD in very small samples, showing feasibility, but with little power to make statements regarding clinical or cognitive effects. Larger, sham‐controlled studies, potentially in subgroups, are necessary to establish whether fNIRS or fMRI neurofeedback training has potential as a treatment for some individuals with ADHD.

Non‐invasive brain stimulation studies have been increasing exponentially over recent years. TMS studies have shown inconsistent findings with the best evidence so far for clinical effects with combined stimulation of DLPFC and IFC with cognitive training (Alyagon et al., 2020). Meta‐analyses of tDCS effects, with the majority of studies targeting left DLPFC, show small effect sizes for improving cognitive functions (Salehinejad et al., 2019; Westwood, Radua, & Rubia, 2021a). Only four studies have tested clinical effects with inconclusive findings. TNS seems to be promising so far in improving ADHD symptoms based on one sham‐controlled RCT, but replication of findings in larger samples is necessary.

However, there are many limitations to the studies conducted, so that strong conclusions cannot be drawn. The majority of stimulation studies have been very small. Most studies only applied one to five stimulation sessions. Furthermore, most studies have used cross‐over designs, which are powerful ways to test within‐subject effects but are confounded by potential longer‐term carry‐over stimulation effects. Given that there is evidence for longer‐term effects of tDCS (Alyagon et al., 2020; Westwood, Radua, & Rubia, 2021a), cross‐over studies may be confounded by these. The gold‐standard method for testing treatments, a double‐blind RCT in parallel groups, has only been conducted in two studies using TMS (Cao et al., 2018; Paz et al., 2018) and three studies using tDCS so far (Cachoeira et al., 2017; Cosmo et al., 2015; Westwood, Criaud, et al., 2021b), with one single‐blind study (Cao et al., 2018). There was a large variability between studies in protocols, such as patient age groups (adults or children), patient inclusion criteria, current density, number of sessions, application of tDCS online or offline, electrodes montage/target area, cathode placement, or combination with cognitive training or not. Furthermore, outcome variables were also highly heterogeneous with use of different clinical and cognitive measures. Variation of any of these factors could have influenced the study results.

There is large heterogeneity on effects of brain stimulation due to variance in skin, skull, or other anatomical parameters that can act as electrical barriers (Antal et al., 2017; Liu et al., 2018). Therefore, the application of one set of parameters for all is unlikely to work and optimal stimulation parameters are likely to differ between individuals. In fact, different participants respond to different intensities as well as frequencies, so that one particular intensity and frequency may be optimal stimulation for some, but over‐ or understimulation for others (Lipka et al., 2021). Age is furthermore likely to have an effect. The skull and cortical surface, which can impede electrical currents, increase over the course of maturation (Mills et al., 2021), which means that the same current intensity for adults is much higher for children and could even have the opposite effect (Kessler et al., 2013; Moliadze et al., 2015). Stimulation parameters that are optimal for adults are hence not easily transferrable to children or adolescents given the developing scalp; and optimal parameters for children are not known so far. There is also evidence that applying a multichannel montage compared to a standard montage can provide more focal stimulation (Fischer et al., 2017). This is furthermore exacerbated in ADHD as it is a heterogeneous disorder with different subgroups with different clinical and neurocognitive profiles (Lambek et al., 2018), which are likely to benefit from different stimulation protocols.

Both for neurofeedback and stimulation studies, the optimal protocols for different age and patient subpopulations need to be systematically tested. Variables to be explored include optimal stimulation/neurofeedback target sites, intensity, frequency and focality of stimulation, duration of stimulation/neurofeedback, frequency of NF/stimulation sessions, and electrode size, inter‐electrode distance, and cathode placement for stimulation. It is thought that brain stimulation combined with cognitive training has a larger potential to improve clinical symptoms and enhance brain plasticity in ADHD than brain stimulation alone. Given the relatively widespread network abnormalities in ADHD, it is also possible that targeting different sites together, such as DLPFC and IFC or parietal sites, may be more successful in improving symptoms than targeting only a single site. Indeed, the most promising stimulation studies that showed clinical improvements were those that targeted both DLPFC and IFC with TMS (Alyagon et al., 2020) or with tRNS (Berger et al., 2021), and both studies combined stimulation with cognitive training.

This also applies to neurofeedback where NF of multivariate patterns that differentiate patients from controls may be more effective than NF of isolated regions of interest (Sato et al., 2013). Multivariate pattern analyses also reflect more ample and spatially sensitive information from the fMRI than traditional methods (Watanabe et al., 2017). Likewise, connectivity‐ or correlation‐based NF of entire neural networks that are affected in ADHD, such as the default mode network, may be more effective in remediating clinical problems than NF of isolated regions (Watanabe et al., 2017).

The control condition is also crucial. For fMRI‐NF it has been debated whether sham NF is the optimal control condition as opposed to alternative region, mental rehearsal, or bidirectional NF control conditions. For studies testing clinical efficacy, comparison with standard treatment (i.e., stimulant medication) or sham NF may be most suitable. Yoked NF has the advantage that it matches the experimental condition on all aspects, except gaining control over the experimental ROI signal, which perfectly controls for motivation and visual stimulation (sham participants get the identical visual stimulation from the active group), and placebo effects, and excludes global effects (Sorger et al., 2019). However, they cannot control for region‐specificity, which would be best tested in NF of an alternative region or bidirectional NF (up‐ and downregulation of the same region), nor can they control for mental strategies used. Applying several control conditions at the same time would be best to address all possible confounds or alternative effects, such as sham‐NF to control for placebo effects, and active control condition to control for site‐specific effects or mental rehearsal for cognitive training effects (Sorger et al., 2019). However, this is impractical and expensive as it would require very large number of subjects (Sorger et al., 2019). For stimulation studies, the cathode position also plays an important role and the electrical current may well be different with different cathode placements even with identical anodal stimulation (Foerster et al., 2018).

Interindividual baseline differences in brain activation and/or cognitive performance have been shown to affect learning of brain self‐regulation or stimulation effects (Krause & Cohen Kadosh, 2014; Lam et al., 2020).

Importantly for ethical reasons, positive or negative side‐effects of regional fMRI‐neurofeedback or stimulation on not self‐regulated/non‐stimulated regions need to be better understood. Stimulation or neurofeedback of specific regions could have a downregulation/downstimulation effect on neighboring regions that are top‐down‐regulated by these regions (in particular when frontal regions are used as targets). Regional stimulation/NF could also negatively affect homologue regions in the opposite hemisphere, which may be indirectly downregulated via interhemispheric inhibition. There is in fact some evidence that stimulating right prefrontal regions could potentially be related to changes in mood in ADHD (Ustohal et al., 2012), which may be related to the fact that the right frontal lobe is more associated with negative emotions (Gainotti, 2018) or that right frontal stimulation may downregulate left frontal lobe activation via interhemispheric inhibition, which could lead to negative mood.

Last but not least, parents tend to prefer neurotherapeutics to medication treatments and parental or participants’ preferences should be taken into account (Buchanan et al., 2022).

Future double‐blind RCTs are needed in large samples to test systematically for effects of different stimulation or neurofeedback protocols on different subpopulations and age groups. While systematic testing could be very costly, this could be made more effective using personalized neuroadaptive Bayesian optimization methods (Lipka et al., 2021).

In conclusion, the substantial knowledge acquired over three decades of fMRI imaging in ADHD has opened up treatment targets for modern neurotherapeutics which seem attractive for children with ADHD due to their safety and minimal side‐effects and their potential for longer‐term neuroplastic effects, compared to medication treatments. However, neurotherapies need to be more thoroughly tested for their short‐ and longer‐term efficacy, optimal “dose” effects (i.e., optimal target site; intensity of stimulation; frequency of stimulation/neurofeedback sessions; differential age‐adjusted protocols), potential costs that may accompany the benefits, and their potential for individualized treatment depending on clinical or cognitive ADHD subtypes. Personalizing treatment based on baseline neurocognitive or brain‐imaging patterns in ADHD, or using machine learning or Bayesian optimization methods (Lipka et al., 2021) is likely more effective for ADHD than a one‐size‐fits‐all approach. It is likely that different clinical or cognitive subgroups of ADHD patients will benefit from either neurofeedback, brain stimulation or medication with individualized protocols and establishing this knowledge will be crucial to the benefit of individual patients.

## CONFLICT OF INTEREST

K.R. has received a grant from Takeda Pharmaceuticals for another project and consultancy fees from Lundbeck and Supernus Pharmaceuticals.
